# Supplementary material for: Identification of photoperiod-induced specific miRNAs in the adrenal glands of Sunite sheep (Ovis aries)
Source: Front Vet Sci. 2022 Jul 22;9:888207. doi: 10.3389/fvets.2022.888207 (PMC9354845; doi:10.3389/fvets.2022.888207)
Supplement: Supplementary file 1 [file Data_Sheet_1.ZIP › Supplementary file/miRDeep2 file.zip!/miRDeep2 file/Novel_Structure/SPLP42A2/Novel_201.pdf]

Provisional ID	: NC_019484.2_43560
Score total	: 2.4
Score for star read(s)	: -1.3
Score for read counts	: 0
Score for mfe	: 2.1
Score for randfold	: 1.6
Score for cons. seed	:
Total read count	: 17
Mature read count	: 13
Loop read count	: 0
Star read count	: 4

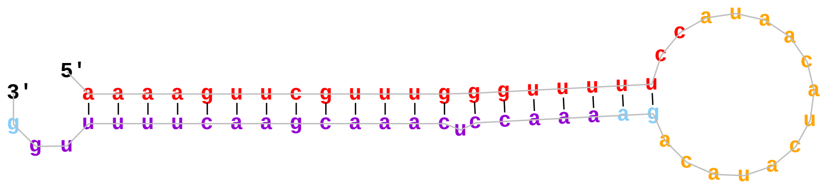

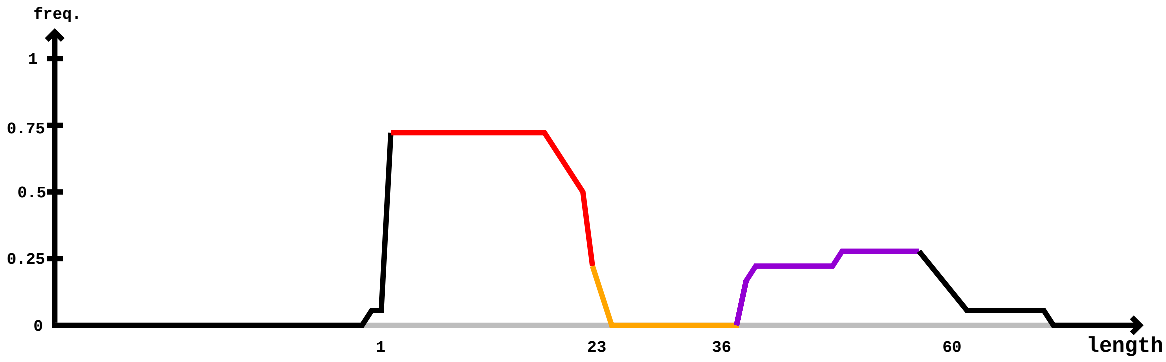

Mature **Star**

```
5'- uuaucagacuggccucuagcuauuggguuggcuaaaaguucguuuggguuuuuccauaacaucauacagaaaaccucaaacgaacuuuuuggccaacccaaugcuacaugg  -3'  obs
    uuaucagacuggccucuagcuauuggguuggcuaaaaguucguuuggguuuuuccauaacaucauacagaaaaccucaaacgaacuuuuuggccaacccaaugcuacaugg       exp
    ............((..((((.((((((((((((((((((((((((((((((((...............))))))).))))))))).))))))))))))))))))))...))  reads  mm  sample
    ...............................cAaaaaguucguuuggguuuuuc.........................................................  1  1  seq
    .................................aaaaguucguuuggguu.............................................................  1  0  seq
    .................................aaaaguucguuuggguuu............................................................  1  0  seq
    .................................aaaaguucguuuggguuuG...........................................................  1  1  seq
    .................................aaaGguucguuuggguuuuu..........................................................  1  1  seq
    .................................aaaaguucguuuggguuuuuA.........................................................  2  1  seq
    .................................aaaaguucgCuuggguuuuuc.........................................................  2  1  seq
    .................................aaaaguucguuuggguuuuucG........................................................  3  1  seq
    .................................aaaaguucguuuggguuuuucAa.......................................................  1  1  seq
    ......................................................................aaaccucaaacgaacuuuuug....................  3  0  seq
    .......................................................................aaccucaaacgaacuuuuuggc..................  1  0  seq
    ................................................................................cgaacuuuuuggccaacccCau.........  1  1  seq
```
